# "The impact of family stress and resilience on child development": the role of parental emotional health and parenting practices in offspring mental health

**DOI:** 10.47626/2237-6089-2024-0794

**Published:** 2025-03-27

**Authors:** Larissa Hallal Ribas, Rafaela Soares Villar

**Affiliations:** 1 Universidade Católica de Pelotas Pelotas RS Brazil Programa de Pós-Graduação em Saúde e Comportamento, Universidade Católica de Pelotas, Pelotas, RS, Brazil.; 2 Universidade Federal de Pelotas Pelotas RS Brazil Programa de Pós-Graduação em Educação, Universidade Federal de Pelotas, Pelotas, RS, Brazil.

The scoping review by Mendes-Sousa et al.^1^ examines the relationship between family stress, child development, and offspring mental health. The review is critical because it revealed that the family environment is an essential determinant of a child's socio-emotional^1^ development and provided us with data to understand this relationship. Since children spend most of their time in the family social environment, this understanding is fundamental for strategies to prevent mental health problems in the offspring.

As main results, we highlight that dysfunctional environments, parental depression, and low parental skills were related to socio-emotional delays in offspring.^1^ In particular, maternal depression was related to developmental delays and internalizing and externalizing symptoms in the child.^1^ Parental mental health is a predictor of a child's mental health,^2^ highlighting the importance of identifying parental psychopathologies, especially in mothers, who are often primary caregivers. Furthermore, Mendes-Sousa et al.^1^ mention that development deficits were greater in the parents’ poor socio-economic and educational status. To profile the caregivers, the authors could have extracted data on social markers such as gender, race, nationality, socioeconomic and cultural status.

Emotional problems in childhood include internalizing symptoms, such as depression and anxiety,^2^ and have been associated with depression in adults.^3^ Behavioral problems, characterized by externalizing reactions such as opposition, inattention, hyperactivity, and aggressiveness,^2^ predicted antisocial tendencies in adulthood.^4^ It would be worth mentioning the sex of the children evaluated in the article, as it can mediate the results since girls may have more emotional problems and boys more behavioral problems.^5,6^

There was also no mention of the reason for evaluating individuals between four and 12 years old. Epigenetics suggests the existence of critical, sensitive childhood periods in which lived experiences can "sculpt" brain development.^7^ School-age is one of these periods,^8^ when new socio-emotional challenges occur, with new interactions and the perception of difficulty in meeting expectations, pressures, and social acceptance. Therefore, children can develop psychopathologies. So, research that identifies risk factors for adverse mental health outcomes at school age is essential.

The caregivers can act as crucial external regulators of children's emotional socialization through guidance, modeling, and setting behavioral expectations. Therefore, more than one caregiver can be important because, in the absence of one due to mental illness, the other can perform this caring role. However, the review shows that not only the presence of caregivers is essential, but also their parenting practices. Children whose parents had poor communication, inhibited emotional expression, insecurity, minimal social involvement, and aggressive discipline tactics had more internalizing and externalizing symptoms.^1^ But positive parenting practices have been linked to better child socio-emotional development.^1^

Finally, the review by Mendes-Sousa et al.^1^ warned about preventing child socio-emotional problems through promoting parental mental health, positive parenting practices, and cohesive family environments. We envision a significant path for subsequent research on maternal emotional overload and the central role of mothers in caring for their offspring, exploring shared care for children and potential public policies aimed at mothers’ mental health and social inclusion.
